# Extended time frame for restoring inner ear function through gene therapy in Usher1G preclinical model

**DOI:** 10.1172/jci.insight.169504

**Published:** 2024-02-08

**Authors:** Ghizlene Lahlou, Charlotte Calvet, François Simon, Vincent Michel, Lauranne Alciato, Baptiste Plion, Jacques Boutet de Monvel, Marie-José Lecomte, Mathieu Beraneck, Christine Petit, Saaid Safieddine

**Affiliations:** 1 Institut Pasteur, Université Paris Cité, Inserm, Institut de l’Audition, Paris, France.; 2 Assistance Publique–Hôpitaux de Paris, Sorbonne University, Service d’ORL et de Chirurgie Cervico-Faciale, DMU ChIR, Paris, France.; 3 Université Paris Cité, Faculté de Médecine, Paris, France.; 4 Université Paris Cité, CNRS UMR 8002, INCC — Integrative Neuroscience and Cognition Center, Paris, France.; 5 Department of Pediatric Otolaryngology, Assistance Publique–Hôpitaux de Paris, Hôpital Necker-Enfants Malades, Paris, France.; 6 Institut Pasteur, INSERM, Pathogenesis of Vascular Infections Unit, Paris, France.; 7 Collège de France, Paris, France.; 8 CNRS, Paris, France.

**Keywords:** Therapeutics, Gene therapy, Mouse models

## Abstract

Neonatal gene therapy has been shown to prevent inner ear dysfunction in mouse models of Usher syndrome type I (USH1), the most common genetic cause of combined deafness-blindness and vestibular dysfunction. However, hearing onset occurs after birth in mice and in utero in humans, making it questionable how to transpose murine gene therapy outcomes to clinical settings. Here, we sought to extend the therapeutic time window in a mouse model for USH1G to periods corresponding to human neonatal stages, more suitable for intervention in patients. Mice with deletion of *Ush1g* (*Ush1g^–/–^*) were subjected to gene therapy after the hearing onset. The rescue of inner ear hair cell structure was evaluated by confocal imaging and electron microscopy. Hearing and vestibular function were assessed by recordings of the auditory brain stem response and vestibulo-ocular reflex and by locomotor tests. Up to P21, gene therapy significantly restored both the hearing and balance deficits in *Ush1g^–/–^* mice. However, beyond this age and up to P30, vestibular function was restored but not hearing. Our data show that effective gene therapy is possible in *Ush1g^–/–^* mice well beyond neonatal stages, implying that the therapeutic window for USH1G may be wide enough to be transposable to newborn humans.

## Abstract 2

In the Usher1g mouse model, the therapeutic window to treat deafness and balance disorders by gene therapy is wider than expected and may be translatable to patients.

## Graphical abstract

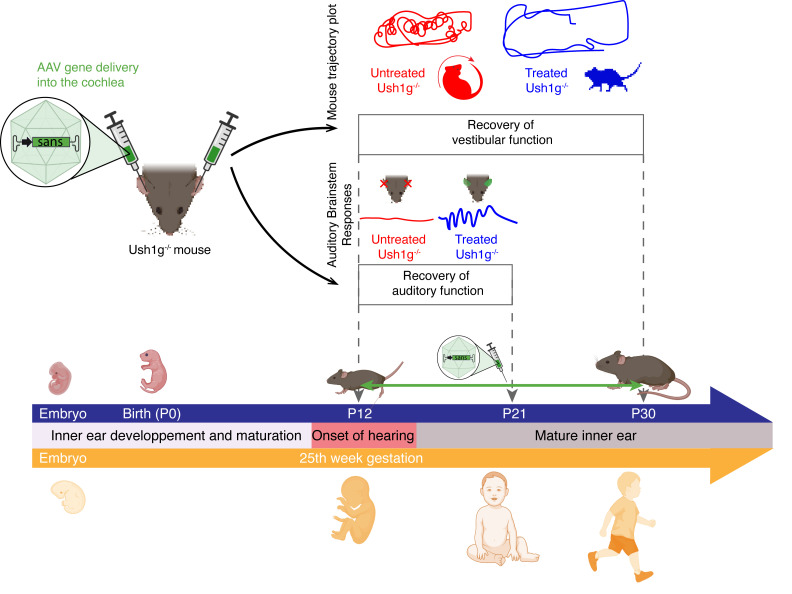

## Introduction

Deafness, with or without associated balance disorders, is the most prevalent inherited sensory disorder in humans, and it is a major public health concern. The clinical prevalence of congenital deafness is about 1 in 700 newborns. About 80% of prelingual deafness cases are attributed to a genetic cause (1). Patients with sensorineural hearing loss are currently fitted with auditory hearing aids if they have mild-to-severe hearing loss and with cochlear implants if they have severe-to-profound deafness. Patients with vestibular disorders currently have limited options, relying on symptomatic medication and vestibular rehabilitation therapy (2), but there is potential for a vestibular implant to become an option in the future, as suggested by Starkov et al. (3). Recent preclinical studies have revealed that gene replacement strategies can have robust and durable therapeutic effects, restoring inner ear function in mouse models of human inner ear diseases (4–9). Such demonstrations have been obtained for several congenital deafness mouse models, including models of User syndrome type I (USH1; Online Mendelian Inheritance in Man #276900), the most severe form of Usher’s deafness-blindness syndrome. USH1 is an autosomal recessive disease characterized by bilateral congenital profound deafness, severe vestibular deficits, and bilateral progressive retinitis pigmentosa leading to blindness (10). Patients with USH1 can benefit from early cochlear implantation, but outcomes are variable (11). The associated vestibular defects lead to a delay in motor acquisitions, gaze instability, and a higher rate of accidental falls in older individuals, which worsens with vision impairment (12). Six genes involved in USH1 have been identified, all encoding proteins involved in the mechanoelectrical transduction of sound vibrations and head motion signals, in the auditory and vestibular hair cells (VHCs), respectively (10). The sensory cells of the auditory organ (the cochlea) and the vestibular organs (utricle, saccule, and the 3 semicircular canals [SCC]) are the hair cells, which harbor a mechanosensory antenna called the hair bundle, consisting of an array of modified microvilli (stereocilia) forming a staircase pattern at their apical surface. The deflection of the hair bundle in response to sound or head movements results in the opening of mechanoelectrical transduction channels located at the tips of the stereocilia. This leads to the conversion of the mechanical signals into electrical signals that are conveyed to the central auditory and vestibular nuclei via the primary auditory fibers forming the VIIIth cranial nerve.

Adeno-associated virus–mediated (AAV-mediated) gene therapy during the neonatal period has proved effective in several mouse models of USH1, including USH1C and USH1G, caused by defects of the genes encoding the scaffold proteins harmonin and sans, respectively (4, 6). Like patients with USH1G, *Ush1g^−/−^* mutant mice are profoundly deaf, with no identifiable auditory brain stem responses (ABRs), and vestibular dysfunctions characterized by locomotor, postural, and gaze stabilization impairments (4, 13). We have shown that sans gene therapy at an early neonatal stage results in a partial rescue of hearing and a robust restoration of vestibular function in *Ush1g^–/–^* mice. However, the neonatal period of mouse inner ear development corresponds in humans to approximately the 19th week of gestation for vestibular function and the 25th week of gestation for auditory function (14, 15). At birth, the vestibular organs and the cochlea of mice are still immature, with the functional onset occurring at about P10 and P12, respectively (16, 17), and their central connections continue to develop well after P12. To establish a transposable therapeutic time window for implementing local gene therapy in the clinical setting for patients with USH1G, we investigated the extent to which such a therapy remains effective beyond P12 (i.e., in the mature inner ear) in a USH1G mouse model.

We found that viral gene replacement therapies applied to the inner ear of *Ush1g^–/–^* mice, even well into the mature stage, substantially rescued the structure and function of both the cochlea and vestibular organs. While the time window for effective treatment of vestibular defects appears to be larger than that for the associated deafness, our results suggest the possibility of extending this time frame enough to be transposable to newborn humans with USH1G, laying the groundwork for future clinical applications.

## Results

### Transduction efficiency in mature inner ear hair cells.

**Figure 1:**
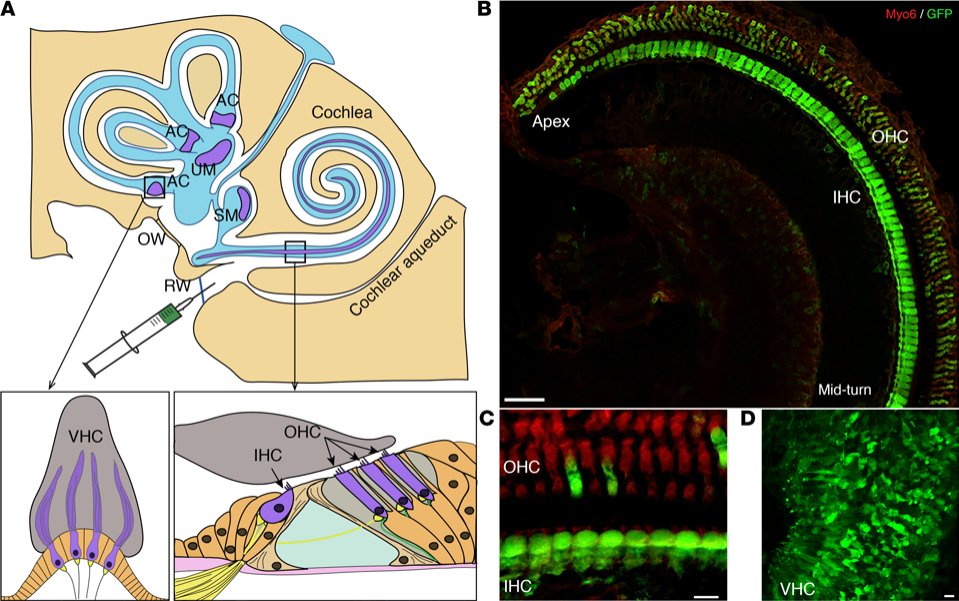
AAV2/Anc80L65 efficiently transduces mature inner ear hair cells. (**A**) The top panel shows a schematic representation of the inner ear (the cochlea and vestibular sensory epithelia in purple), illustrating viral vector injection through the round window membrane (RWM) of the cochlea. The vestibular hair cells (VHCs) are located in the crista ampullae (AC) of each semicircular canal and in the utricular and saccular macula (UM and SM) (left), whereas the sensory hair cells of the cochlea, inner hair cells (IHCs) and outer hair cells (OHCs), are harbored in the organ of Corti (right). OW, oval window; RW, round window membrane. (**B**) Organ of Corti, spanning from the middle to apical turns of the cochlea in a wild-type mouse, underwent AAV2/Anc80L65-CMV-GFP injection on P20 and was immunostained for myosin 6 (in red) and GFP (in green) on P25. (**C** and **D**) Maximum-intensity projections of confocal *z*-sections of IHCs from the cochlear middle turn (**C**) and SM (**D**). Almost all IHCs and most VHCs were transduced with AAV2/Anc80L65. Scale bars: 50 μm (**B**); 10 μm (**C** and **D**).

In the inner ear, the cellular tropism and transduction efficacy of recombinant AAV vectors vary depending on serotype, the promoter used, route of administration, and stage of development (18–21). We have previously observed that there is a positive correlation between the transduction rate of the inner ear sensory cells and the level of functional restoration (4). It is, therefore, essential to target the largest possible number of inner ear cells within a given therapeutic time window in order to optimize gene therapy outcomes. We used the ancestral AAV Anc80L65 vector, which has been shown to efficiently transduce the sensory hair cells of the mature inner ear (18, 22, 23). Due to interstudy variability in transduction efficiency for a given AAV, depending on promoters, transgenes, vector doses, and mode of purification as well as age of administration, we wanted to check these settings in-house for the AAV2/Anc80L65. To this end we assessed the hair cell transduction efficiency of this serotype expressing GFP as reporter gene under the control of the CMV promoter. The viral preparation was administered through the round window membrane (RWM) in C57BL/6 wild-type mice on P20 (Figure 1A). The inner ear organs (organ of Corti, SCC crista ampullaris, and utricle and saccule maculae) were microdissected and immunolabeled for myosin VI to stain the inner ear hair cells and for GFP to visualize the transduced cells. AAV2/Anc80L65 transduced both cochlear hair cells and VHCs, but the transduction rate varied with cell type (Figure 1, B–D). The mean transduction rate was 97% ± 6% (transduction range, 83%–100%) for inner hair cells (IHCs) and 13% ± 24% (transduction range, 0%–61%) for outer hair cells (OHCs) (*n* = 7, Figure 1, B and C). Transduction rates for VHCs were 45% ± 24% (range, 11%–90%) in the SCC crista ampullaris (*n* = 2, Figure 1D), and 57% ± 17% (range, 31%–90%) in the utricle macula. The hair cell transduction rates observed in this study confirm that AAV2/Anc80L65 remains one of the most effective variants for transducing sensory cells in the adult inner ear to date, making it the most appropriate serotype for this therapeutic window study.

### Sans gene therapy restores protein targeting and rescues hair cells from degeneration in the inner ear of adult Ush1g^–/–^ mice.

**Figure 2:**
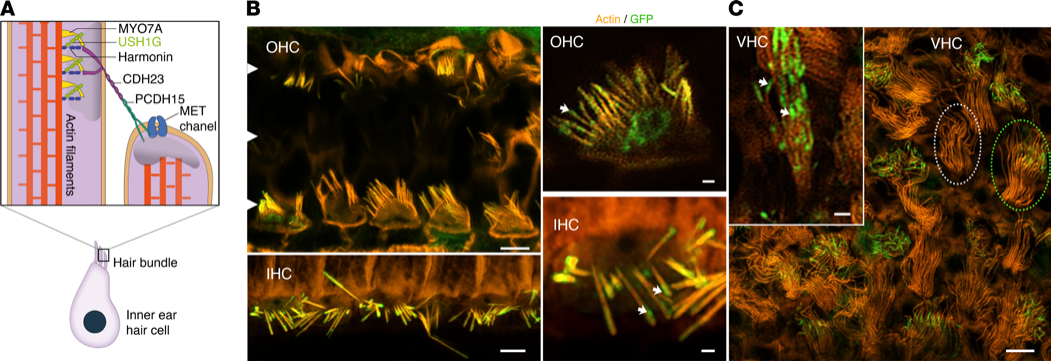
AAV2/Anc80L65 vector-mediated transfer of *Sans* cDNA fused to GFP restores protein expression and targeting. (**A**) Schematic representation of the mechanoelectrical transduction (MET) machinery in the hair bundle of inner ear hair cells. The Ush1g protein (Sans protein) is located in the upper tip link density, together with the myosin 7A (MYO7A) and the harmonin. The tip link connecting 2 stereocilia is composed of the cadherin23 (CDH23) and the protocadherin15 (PCDH15) proteins. White arrowhead points the three rows of OHCs. (**B** and **C**) Confocal images of auditory (**B**) and vestibular (**C**) hair cells from P32 *Ush1g^–/–^* mice following intracochlear injection of AAV2/Anc80L65-CMV-GFP-*Sans*-WPRE at P18, immunolabeled for actin (in orange) and GFP (in green), showing that the GFP-*Sans* exhibits a localization pattern mirroring the typical distribution of the protein Sans in the transduced inner hair cells (IHCs), outer hair cells (OHCs), and vestibular hair cells (VHCs). GFP-*Sans* is localized at the tip of the stereocilia (white arrow). The dotted circles delineate transduced (green) and nontransduced (white) VHCs. Scale bars: 5 μm (low-magnification images); 1 μm (high-magnification images).

We wanted to establish the precise targeting of the virally delivered protein Sans to the top of the stereocilia of the mature cochlea. However, despite numerous attempts to remanufacture the Sans antibody we previously characterized (4), and the commercial one from Abcam (catalog ab150820), a nonspecific signal persisted in inner ear hair cells of adult *Ush1g^–/–^* mice using these antibodies. This nonspecific signal hindered the direct localization of the exogenous Sans protein delivered by the AAV. We tackled this challenge indirectly by creating a viral preparation with an identical configuration, producing Sans fused to GFP (AAV2/Anc80L65-CMV-GFP-*Sans*-WPRE, 6.2 × 10^12^ gc/mL). The viral preparation was administered through the RWM of P18 *Ush1g^–/–^* mice (*n* = 4) and *Ush1g^+/-^* mice (*n* = 4). Two weeks after the injection, the sensory epithelia of the cochlea and vestibular organs were microdissected and immunolabeled for actin and GFP. Interestingly, GFP showed a primarily cytoplasmic distribution when produced on its own (see Figure 1). However, when fused to Sans, GFP underwent a relocation to the tips of IHC and OHC stereocilia. This mirrors the typical Sans localization, indicating effective protein targeting after intracochlear injection in *Ush1g^–/–^* mice (Figure 2). In addition, the transduction profile closely matched that obtained with AAV2/Anc80L65-CMV-GFP. As expected, the transduction of OHC was lower compared with that of IHC (respectively, 25% ± 12.2% [range, 1%–56%] and 97% ± 1.9% [range, 93%–100%], *n* = 4). Transduction of VHC was variable and lower for cristae ampullae compared with the utricle maculae (respectively, 34% ± 11.7% [range, 8%–56%] and 49% ± 12.6% [range, 26%–85%], *n* = 4).

**Figure 3:**
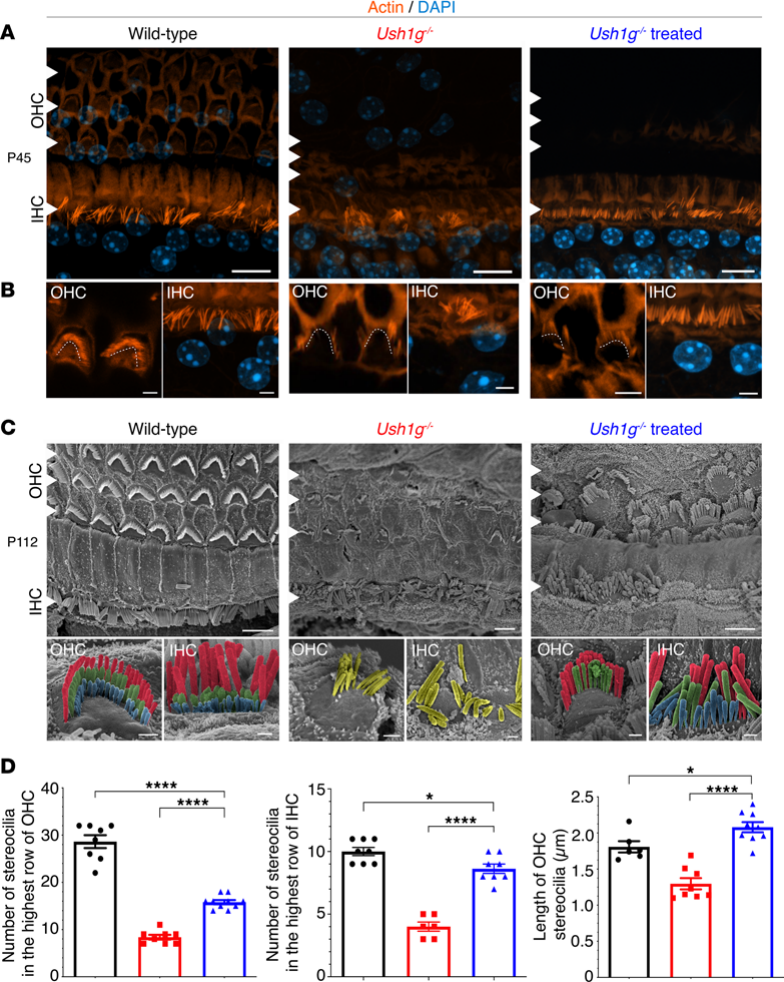
Viral vector-mediated transfer of the *Sans* cDNA at a mature stage restores cochlear hair cell architecture. (**A**) Confocal images of auditory hair cells from P45 wild-type (left), untreated *Ush1g^–/–^* (middle), and treated *Ush1g^–/–^* (right) mice, immunolabeled for actin (in orange). DAPI was used to stain the nuclei (in blue). White arrowheads points the three rows of the outer hair cells (OHCs) and one row of inner hair cells (IHCs). Scale bars: 10 μm. (**B**) A close-up view of the outer hair cells (OHCs) and inner hair cells (IHCs) at high magnification, showcasing the recovery of a near-normal stereocilia shape in the treated mice. Scale bars: 3 μm. (**C**) Low- and intermediate-magnification scanning electron micrographs of the organ of Corti of wild-type (left), untreated *Ush1g^–/–^* (middle), and treated (at right) *Ush1g^–/–^* mice, showing a partial restoration of hair-bundle architecture in IHCs and OHCs. Scale bars: 50 μm (low-magnification images); 1 μm (intermediate-magnification images). (**D**) Comparative analyses of stereocilia number and length in the tallest row in both OHCs and IHCs showing a significant improvement in *Ush1g^–/–^* mice after gene therapy treatment (blue) relative to untreated mice (red), the measurements for wild-type mice are shown in black (1-way ANOVA). Number of stereocilia in the higher row of OHCs and IHCs and length of OHC stereocilia were assessed, respectively, in 10, 8, and 9 treated *Ush1g^–/–^* mice; 8, 6, and 8 untreated *Ush1g^–/–^* mice; and 10, 8, and 6 wild-type mice. *P < 0.05 and ****P < 0.0001.

**Figure 4:**
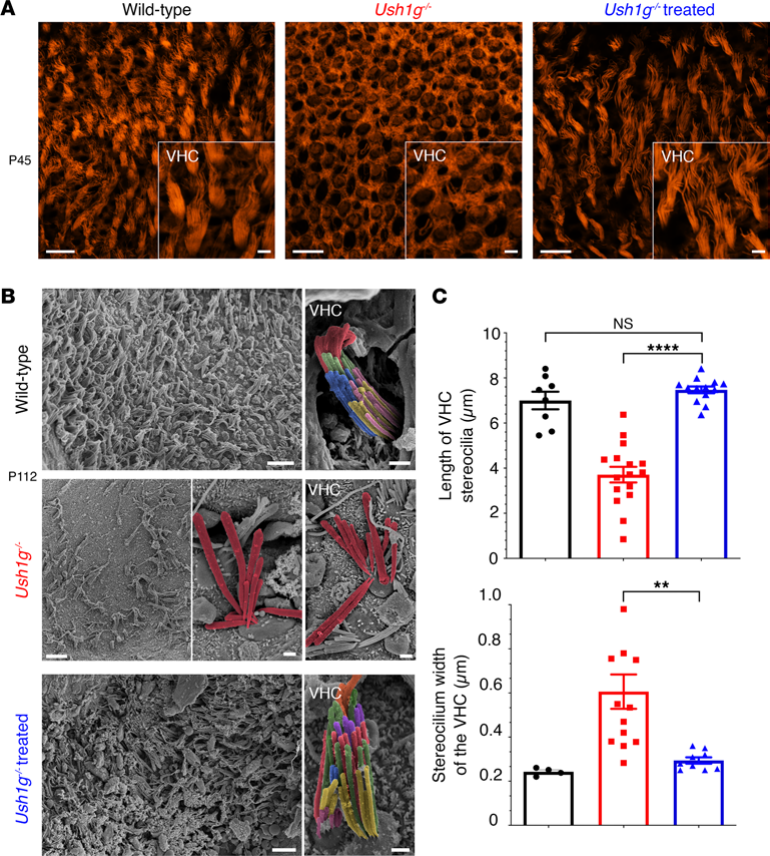
Viral vector-mediated transfer of *Sans* cDNA restores vestibular hair cell architecture. (**A**) Low- and high-magnification (inset) confocal microscopy images of utricular hair cells from P45 wild-type (left), untreated *Ush1g^–/–^* (middle), and treated (right) *Ush1g^–/–^* mice, immunolabeled for actin (in orange), demonstrating that the vestibular sensory epithelium of the treated *Ush1g^–/–^* mouse is populated with hair cells with stereocilia of nearly normal shape, similar to those in the wild-type mouse. In contrast, the vestibular epithelium of the untreated *Ush1g^–/–^* mouse is entirely devoid of hair cells with stereocilia. Scale bars: 10 μm (low-magnification images); 5 μm (high-magnification images). (**B**) Low- and high-magnification scanning electron micrographs of the utricular sensory epithelium of P112 wild-type (top), untreated *Ush1g^–/–^* (middle), and treated *Ush1g^–/–^* (bottom) mice. These results show that gene therapy treatment prevents vestibular hair cell (VHC) degeneration and restores the staircase pattern of hair bundles. (**C**) Comparative analyses of the hair cell stereocilia on P40, with treated *Ush1g^–/–^* mice displaying a full recovery of stereocilia length (blue), compared with wild-type mice (black) and untreated *Ush1g^–/–^* mice (red) (top, *P* < 0.0001, 1-way ANOVA; *n* = 13, 16, and 8, respectively, for treated *Ush1g^–/–^*, untreated *Ush1g^–/–^*, and wild-type mice). On P112, considerable heterogeneity in VHC stereocilium diameter is observed in untreated *Ush1g^–/–^* mice; this heterogeneity was significantly decreased after gene therapy treatment (bottom, *P* = 0.009, 1-way ANOVA; *n* = 9, 13, and 4, respectively, for treated *Ush1g^–/–^*, untreated *Ush1g^–/–^*, and wild-type mice). **P < 0.01 and ****P < 0.0001.

We sought to define a transposable therapeutic time window applicable to patients with USH1G by investigating the outcomes of gene replacement therapy performed during the functional maturation of auditory and vestibular functions, namely between P12 and P30, in *Ush1g^–/–^* mice. The structural damage to the inner ear hair cell stereocilia is already severe in these mice at P2.5 (4), and it worsens during cochlear development (13). An AAV2/Anc80L65-CMV vector driving Sans expression (AAV2/Anc80L65-CMV-*Sans*-WPRE 5.15 × 10^12^ gc/mL, Gene Transfer Vector Core) was engineered to assess its therapeutic effect after injection through the RWM into the inner ear of *Ush1g^–/–^* mice at P14 (*n* = 4). The sensory epithelia of the cochlea and vestibular organs were microdissected 4 weeks after the injection and immunolabeled for actin (Figure 3 and Figure 4). Confocal microscopy imaging showed the stereocilia to be abnormal or absent in the IHCs, OHCs, and VHCs in the untreated *Ush1g^–/–^* mice. By contrast, the stereocilia were well organized in the treated mice, in particular in IHCs and VHCs, in which they displayed staircase patterns similar to those characteristic of wild-type hair bundles. Thus, the virally driven expression of the Sans protein resulted in an effective restoration of stereocilia architecture in these cells (Figure 3, A and B, and Figure 4A). Likewise, scanning microscopy examination confirmed that the hair bundles of inner ear hair cells in *Ush1g^–/–^* mice underwent fragmentation and degeneration starting from P2.5 and worsening with age (4). At P22, the stereocilia of both IHCs and OHCs are fewer in number and shorter throughout the cochlear spiral than those of their wild-type counterparts, while the VHC stereocilia appear collapsed (4, 13). From P22 onward, the hair cell stereocilia in both the cochlea and vestibular end organs underwent degeneration in a spreading process that became more and more severe throughout the epithelium by P40 (Supplemental Figure 1; supplemental material available online with this article; https://doi.org/10.1172/jci.insight.169504DS1). On P100, entire areas of the auditory and vestibular sensory epithelia appeared devoid of hair cells, indicating that, at this stage, the degeneration process also affects the sensory cells. The stereocilia of the few remaining VHCs had a highly abnormal morphostructure (Supplemental Figure 1). Scanning microscopy examinations of the inner ears of treated *Ush1g^–/–^* mice on P112 showed that gene therapy had prevented cochlear hair cell degeneration and restored stereocilia to a near-normal shape (Figure 3, B and C). The rescued OHCs had a significantly larger number of stereocilia in the tallest row compared with untreated *Ush1g^–/–^* mice (Figure 3D; 16 ± 0.4 stereocilia, *n* = 10 cells, vs. 8 ± 0.5 stereocilia, *n* = 8 cells, respectively, *P* < 0.0001, 1-way ANOVA). Furthermore, the tallest OHC stereocilia were of almost normal length: 2.1 ± 0.07 μm for treated *Ush1g^–/–^* mice (*n* = 9 cells) and 1.8 ± 0.08 μm (*n* = 6 cells) for wild-type mice (*P* = 0.02, 1-way ANOVA), whereas the tallest OHC stereocilium length was reduced to 1.3 ± 0.08 μm in untreated *Ush1g^–/–^* mice (*n* = 8 cells; *P* < 0.0001, 1-way ANOVA). The number of stereocilia in the tallest row of IHC was also significantly higher in treated *Ush1g^–/–^* mice compared with that in untreated mice (9 ± 0.4 stereocilia, *n* = 8 cells, vs. 4 ± 0.4 stereocilia, *n* = 6 cells, *P* < 0.0001, 1-way ANOVA), and was close to that in wild-type mice (10 ± 0.3 stereocilia, *n* = 8 cells, *P* = 0.01, 1-way ANOVA).

Similarly, the stereocilia of the VHCs in the utricular macula of the treated mice had a near-normal structure with a typical staircase pattern (Figure 4, B and C). At P40, the length of the tallest VHC stereocilia in treated *Ush1g^–/–^* mice was similar to that in wild-type mice (7.5 ± 0.15 μm, *n =* 13 cells, vs. 7 ± 1.39 μm, *n =* 8 cells, respectively, *P* > 0.05, 1-way ANOVA), whereas the VHC stereocilia in untreated *Ush1g^–/–^* mice were significantly shorter (3.7 ± 0.35 μm, *P* < 0.0001, 1-way ANOVA, *n* = 16). At P112, a near-complete restoration of stereocilium diameter relative to that in untreated *Ush1g^–/–^* mice was observed (0.29 ± 0.01 μm, *n =* 9 cells, vs. 0.6 ± 0.08 μm, *n =* 13 cells, in treated and untreated *Ush1g^–/–^* mice, respectively, *P* = 0.009, 1-way ANOVA). However, the hair cell stereocilia had similar abnormal appearance in the crista ampullaris of treated and untreated mice. This is not a surprising finding, since the transduction rate of hair cells in the SCC sensory epithelia was lower than that observed in the utricular macula. Of note, the hair bundle morphological aspect of the contralateral inner ear was similar to that of the untreated *Ush1g^–/–^* mice indicating little to no diffusion of the viral particles to the right ear. Additional morphological and structural analyses are provided in Supplemental Table 1.

### Time window for gene therapy to restore hearing in Ush1g mice.

**Figure 5:**
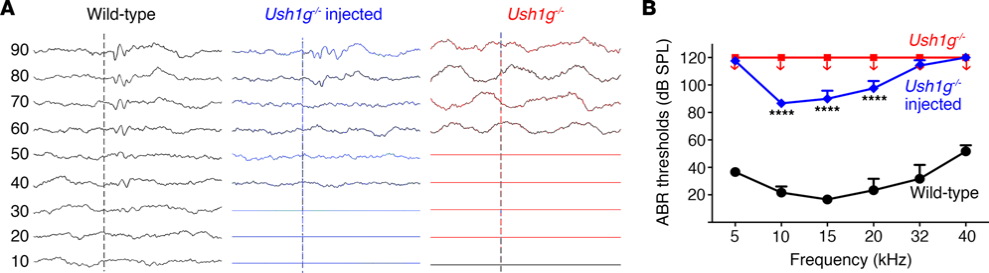
AAV2/Anc80L65-*Sans* gene therapy at the P12–P21 stage restores hearing in *Ush1g^–/–^* mice. (**A**) Auditory brain stem response (ABR) traces for 15 kHz stimulation in wild-type, treated *Ush1g^–/–^*, and untreated *Ush1g^–/–^* mice. (**B**) ABR thresholds in P40 wild-type (*n* = 3), untreated *Ush1g^–/–^* (*n* = 5), and treated *Ush1g^–/–^* mice (*n* = 12), showing a partial recovery for the 10, 15, and 20 kHz frequencies (2-way ANOVA). ****P < 0.0001.

To assess the window for effective restoration of hearing function by gene therapy in *Ush1g^–/–^* mice, ABR recordings were performed at adult stages (P40 and P60) in response to tone bursts at frequencies of 5, 10, 15, 20, 32, and 40 kHz on untreated mice and on mice treated between P12 and P21 (*n* = 36) or at a later stage (P22–P30, *n* = 11). While the untreated *Ush1g^–/–^* mice were all found to be profoundly deaf, about one-third of mice (*n* = 12) treated between P12 and P21 showed a significant restoration of auditory function on P40, with clearly identifiable ABR waves for frequencies between 10 and 20 kHz and sound levels of 70 dB SPL or greater (*P* < 0.0001, 2-way ANOVA; Figure 5). Notably, this partial rescue persisted up to P60 (Supplemental Figure 2). However, no hearing restoration was achieved when gene therapy was administered after P21 (0 of 9 treated ears after P21), suggesting that P21 may constitute an upper limit for the restoration of hearing with this recombinant viral vector configuration. Of note, there was no correlation between the level of auditory threshold restored and the age of mice treated between P12 and P21 (Supplemental Figure 3A).

### Time window for gene therapy to restore vestibular function in Ush1g mice.

**Figure 6:**
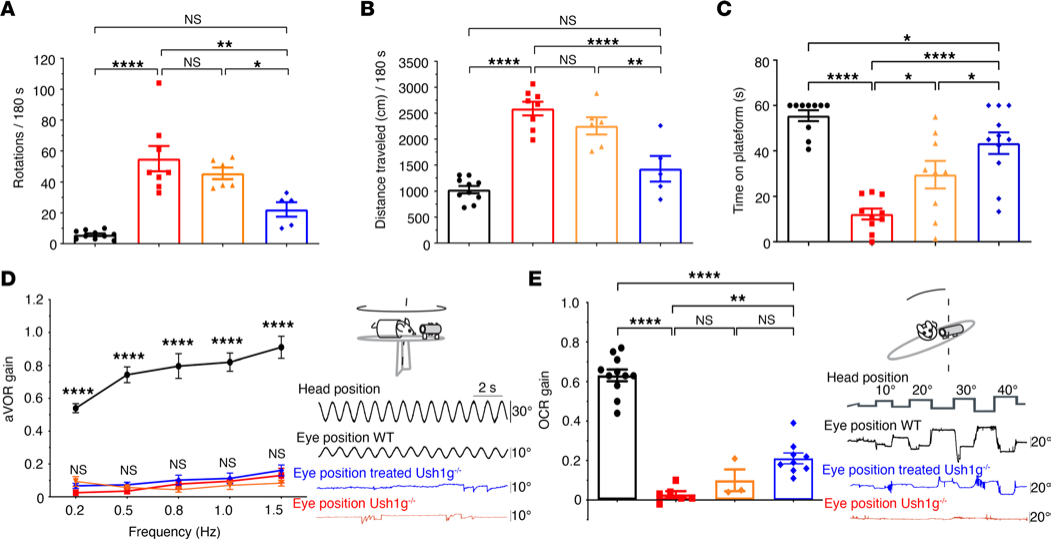
AAV2/Anc80L65-*Sans* gene therapy at the P12–P30 stage restores the balance function in *Ush1g^–/–^* mice. (**A**–**C**) Locomotor tests performed at P40, comparing wild-type (black), untreated *Ush1g^–/–^* (red), and treated *Ush1g^–/–^* mice after unilateral (orange) or bilateral (blue) AAV2/Anc80L65-*Sans* injection. (**A**) Video tracking, performed on 5, 6, 8, and 10 bilaterally, unilaterally treated, untreated *Ush1g^–/–^*, and wild-type mice, respectively, showed a significant improvement after bilateral injection in circling behavior *(P* = 0.001, 1-way ANOVA) and (**B**) distance traveled (*P* < 0.0001, 1-way ANOVA) relative to untreated *Ush1g^–/–^* mice. (**C**) In the platform test, *Ush1g^–/–^* mice subjected to unilateral (*P* = 0.019, 1-way ANOVA, *n* = 9) or bilateral (*P* < 0.0001, 1-way ANOVA, *n* = 11) injections of gene therapy agent spent a longer time on the platform than untreated mice (*n* = 10); 9 wild-type mice were analyzed. (**D** and **E**) Angular vestibulo-ocular reflex gain (aVOR; **D**) and static ocular counter roll gain (OCR; **E**), determined by video-oculography after sinusoidal horizontal rotation (at different frequencies) and static head tilt roll, at P70, on 9, 3, 4, and 11 bilaterally treated (blue), unilaterally treated (orange), untreated *Ush1g^–/–^* (red), and wild-type mice (black), respectively. Static OCR gain was significantly greater after bilateral injection than in untreated *Ush1g^–/–^* mice (*P* = 0.0063, 1-way ANOVA). *P < 0.05, **P < 0.01, ****P < 0.0001.

We evaluated the vestibular phenotype of untreated and treated *Ush1g^–/–^* mice with various behavioral tests performed at least 15 days after treatment (24). We previously showed that restoration of vestibular function to wild-type levels was obtained when unilateral gene therapy was administered at the neonatal stage (4). By contrast, unilateral treatment at later stages (P12–P30) led only to a partial and rather modest recovery (Figure 6, A–C). We hypothesized that this partial rescue was due to a lower rate of VHC transduction, and to the lack of diffusion of the recombinant vector to the contralateral ear in adult mice, as it occurs in neonatal mice through the cochlear aqueduct and the cerebrospinal fluid (18, 25) (Supplemental Figure 4). To verify this hypothesis, we assessed the restoration of vestibular function in *Ush1g^–/–^* mice subjected to a bilateral injection at mature stage (P13–P25) using the behavioral tests mentioned above. The results of all these tests indicated a significantly improved rescue of locomotor and vestibular balance functions in these mice (Figure 6). No correlation was observed between the rescue effect and the age of mice at injection, treated either unilaterally or bilaterally (Supplemental Figure 3, B and C).

In balance platform tests performed on P40 (Figure 6C), the untreated *Ush1g^−/−^* mice spent much shorter times on the platform (mean, 12 ± 2.3 s; range, 0–22 s; *n* = 10) than mice that had received a unilateral injection (mean, 30 ± 5.9 s; range, 1–55 s; *n* = 9; *P* = 0.01, 1-way ANOVA), but those subjected to bilateral injections sustained their balance on the platform for the longest times (mean, 43 ± 4.8 s; range, 13–60 s; *n* = 11; *P* < 0.0001, 1-way ANOVA). In the contact righting test, none of the untreated *Ush1g^–/–^* mice tested (*n* = 0 of 10) managed to roll over onto their feet, whereas the majority of mice that had received injections of the gene therapy agent (unilateral, *n* = 7 of 9, *P* = 0.002; bilateral, *n* = 9 of 11, *P* = 0.0008, χ^2^ test) displayed almost wild-type behavior. Similarly, all the untreated *Ush1g^–/–^* mice (*n* = 10) curled their trunks toward their tails, whereas most of the treated mice reached or tended to reach the horizontal surface (unilateral, *n* = 8 of 9, *P* = 0.0005; bilateral, *n* = 9 of 11, *P* = 0.0008, χ^2^ test). All behavioral analysis is detailed in Supplemental Table 2.

In addition, while all of the untreated *Ush1g^–/–^* mice displayed head bobbing and circling (10 of 10 mice), such behaviors were observed significantly less frequently in the treated mice (unilateral injection, *n* = 4 of 9, *P* = 0.01; bilateral injection, *n* = 4 of 11, *P* = 0.004, Fisher’s exact test). Circling was seen in 5 of 9 and 5 of 11 mice after unilateral and bilateral injections, respectively (*P* = 0.03 and *P* = 0.01, respectively, Fisher’s exact test). Video tracking on P40 showed that the number of rotations over a period of 180 seconds was significantly lower in mice that had received bilateral injections (mean, 22 ± 4.7; range, 10–33, *n* = 5) than in untreated mice (mean, 55 ± 8.2; range, 33–104, *n* = 8; *P* = 0.001, 1-way ANOVA; Figure 6A), or in mice that had unilateral injections (mean, 46 ± 3.7; range 36–56, *n =* 6; *P* = 0.03, 1-way ANOVA).

Mice that had received bilateral injections also covered a much shorter distance in 180 seconds (mean, 1,430 ± 244.8 cm; range, 840–2,260 cm) compared with untreated mice (mean, 2,590 ± 131.8 cm; range, 1,984–3,064 cm; *P* < 0.0001, 1-way ANOVA; Figure 6B and Supplemental Figure 5) or mice that had received unilateral injections (mean, 2,255 ± 165.6 cm; range, 1,767–2,883 cm; *P* = 0.003, 1-way ANOVA).

Finally, during the swimming test, all the untreated *Ush1g^–/–^* mice (*n* = 10) started to drown and had to be immediately rescued, whereas a few (2 of 9) of the mice receiving unilateral injections and the majority (6 of 11) of those receiving bilateral injections were able to swim. The recovery of vestibular function after bilateral injections persisted at least until P100 (Supplemental Figure 6).

In order to assess specific vestibular recovery and distinguish between canal- and otolith-dependent functions, we used video-oculography on P70 to investigate the vestibulo-ocular reflex (VOR) in untreated *Ush1g^–/–^* mice (*n* = 4), *Ush1g^–/–^* mice receiving bilateral injections (*n* = 9) between P15 and P17, and wild-type mice (*n* = 11). Due to the modest recovery obtained after unilateral injection, only 3 *Ush1g^–/–^* mice receiving unilateral injection were tested, and all showed a modest nonsignificant recovery in VOR (Figure 6, D and E). During spontaneous nystagmography (in absence of vestibular stimulation) on P70, gaze was completely stable in all wild-type mice, whereas pupil flutter was observed in all of the untreated *Ush1g^–/–^* mice and unilaterally treated mice. However, most (8 of 9) of the treated *Ush1g^–/–^* mice had unilateral spontaneous nystagmus, suggesting that the restoration of vestibular function was likely asymmetric in most individuals. Of note, no eye fluttering was observed in 1 bilateral treated mouse, indicating that complete gaze stability can be recovered following bilateral injection at mature stage.

In agreement with the observed low hair cell transduction rate in the SCC sensory epithelia, angular VOR gain, which is used to explore horizontal canal function, was absent at all frequencies studied (0.2, 0.5, 0.8, 1, and 1.5 Hz), in both treated and untreated *Ush1g^–/–^* mice (Figure 6D).

Remarkably, static utricular function was partially restored after bilateral gene therapy, with a significant increase in static ocular counter roll (OCR) gain in the bilaterally treated *Ush1g^–/–^* mice relative to that in untreated *Ush1g^–/–^* mice (0.21 ± 0.03 [range, 0.11–0.39], *n* = 9, vs. 0.03 ± 0.03 [range, 0.00–0.10], *n* = 4, *P* = 0.0063 – 2-way ANOVA), although the wild-type OCR gain of 0.61 ± 0.09 [0.44–0.75] (*n* = 11) was not reached (Figure 6E). The increase in OCR gain remained stable at P140 (Supplemental Figure 7).

These data, together with the structural improvement in VHC, demonstrate a significant rescue of utricle-dependent vestibular function by bilateral injections, associated with a substantial recovery of otolith-dependent gaze stabilization and improvements in balance and locomotor capacities.

## Discussion

Despite ongoing exciting advances in applying the translational gene therapy approach to mouse models of human deafness, many challenges lie ahead before this approach can be used to treat inner ear diseases in humans (26). The preclinical investigations performed so far demonstrate the feasibility and efficacy of gene therapy for restoring hearing and balance in several mouse models of genetic inner ear defects (4–9, 27, 28). In most of these studies, the gene therapy was carried out during the neonatal period in mice. This would correspond in humans to a time window falling between 18 and 25 weeks of gestation, implying interventions in utero. To be feasible, the clinical application of gene therapy for hearing and vestibular defects in humans will require the development of treatments that are effective when administered to mouse models at a stage that corresponds to human neonatal period. Here, we assessed the efficacy of gene therapy performed after the onset of hearing in mice (P12) in a mouse model of USH1G. We previously showed that virus vector-mediated gene therapy in neonatal *Ush1g*^–/–^ mice results in a partial restoration of hearing and a long-lasting, almost complete recovery of vestibular function (both otolithic and SCC function). The partial restoration of hearing was presumably due to the very low transduction rate of OHCs (4). Our strategy here was to deliver the Sans-encoding cDNA with AAV2/Anc80L65, a serotype that has been shown to efficiently transduce mature inner ear hair cells (23). We found that unilateral viral gene therapy in *Ush1g^–/–^* mice at a mature stage prevented cochlear hair cells and VHCs from degeneration, and reestablished the characteristic staircase organization of their stereociliary hair bundles. Interestingly, the rescue of morphostructure was more robust for IHCs than OHCs in the cochlea, whereas it was more evident in utricular than in ampullary VHCs. This finding is consistent with the differences in hair cell transduction rate observed after injection through the RWM, resulting in high transduction rates of IHCs, but a lower and variable transduction rate of OHCs and VHCs, especially in the SCC crista ampullae at mature stages.

The improvement in cochlear hair cell structure, mainly IHCs, led to a partial rescue of hearing, which persisted until at least P60 when AAV2/Anc80L65-CMV-*Sans*-WPRE was administered between P12 and P20. No hearing restoration was observed for treatment performed later than P21. This stage thus indicates an upper limit for the therapeutic time window beyond which gene therapy would be ineffective in the *Ush1g^–/–^* mouse model. The partial improvement of hearing observed probably indicates that the number of transduced OHCs — the functions of which are essential for cochlear amplification and normal auditory function — remains below that required to fully rescue hearing. Indeed, in mouse models of DFNA25 and DFNB9, which are both profoundly deaf and where the IHCs are defective but the OHCs are functional, gene therapy administered at a mature stage restored hearing to near-normal thresholds (5, 28). This finding suggests that improvements in the efficiency of OHC transduction are paramount for the restoration of normal hearing thresholds in deafness affecting both IHCs and OHCs function. This conclusion is consistent with the reported positive correlation between the rescue of hearing threshold and the percentage of sensory cells transduced within the inner ear (4). It may, therefore, be possible to rescue the inner ear function of *Ush1g^–/–^* mice to wild-type levels by using an AAV serotype that transduces OHCs more efficiently. Unfortunately, the OHCs of the mature cochlea have proved resistant to transduction with all AAV serotypes tested to date (18, 22, 23, 29, 30).

Our results also show that the bilateral delivery of AAV2/Anc80L65-CMV-*Sans*-WPRE to the inner ears of *Ush1g^–/–^* mice at a mature stage induces significantly greater improvements in vestibular behavior than the unilateral delivery (Figure 6, A–C). Gene therapy administered at neonatal stages resulted in an improvement in vestibular behavior to wild-type levels (4). The long-term restoration of locomotor function to near-normal levels observed in mice treated at neonatal stages is due to the high rate of VHC transduction in both ears, probably reflecting contralateral leakage of the viral preparation (Supplemental Figure 4). By contrast, very few of the contralateral inner ear cells were transduced following unilateral gene therapy at a later stage (18), at which VHC transduction rates were also much lower.

Remarkably, bilateral gene therapy carried out as late as P30 led to significant utricular recovery in *Ush1g^–/–^* mice, suggesting that the time window for gene therapy is broader for vestibular deficits linked to USH1G than for the associated deafness. Interestingly, a significant restoration of many locomotor-related behaviors was observed, despite static head tilt analysis showing restoration of only one-third of the OCR gain. Since there was no recovery of canal-related function, our results confirm that otolithic organs, encoding the head-in-space position relative to gravity, provide an essential contribution for balance, postural control, and locomotion (17). We have previously shown that mice with only two-thirds of the normal OCR gain conserve normal swimming ability, while animals without otolith function are unable to swim (31). This suggests that, even with partial restoration, the redundancy of hair cell directions in the macula, taking into account hair cells with opposite axes on either side of the curved striola (in both ears), could compensate for hair cell loss. Our results seem to confirm this, as performance in balance and locomotion tests exceeded expectations, given the absence of improvements in angular VOR gain in mice treated with bilateral injections. This finding is very encouraging for gene therapy in humans, as even if transduction rates are low, a partial restoration of otolithic organ function may be of great benefit for the development of children with USH1.

In conclusion, our study has established the upper limit of the time window during which gene therapy can effectively restore hearing and vestibular functions in *Ush1g* mice, with extensions up to P21 and P30, respectively. It is essential to emphasize that the observed restoration of auditory and vestibular functions in *Ush1g* mice suggests that the exogenous Sans protein, administered to these mice during mature stages, has the capability to integrate into the Usher1 network, crucial for hearing and balance functions (32). Therefore, AAV vector-based gene therapy strategies targeting the near-mature inner ear can significantly rescue the structure and function of both auditory and vestibular organs. Considering that a 21-day-old mouse represents a therapeutic window in humans similar to that of a young individual aged 3–4 years, these findings establish a transposable therapeutic time window for newborn humans and lay the foundations for future clinical applications (33). Indeed, today, systematic screening for hearing loss in newborns, which is widely adopted throughout the world, has improved the early diagnosis and intervention in neonatal patients as well as time to intervention.

## Methods

### Viral vector construct and packaging.

The p0101_CMV-SV40-*Sans*-bGH plasmid was generated by amplifying the murine *Sans* cDNA sequence (GenBank no. NM_176847) by PCR (1,401 bp amplicon, with 5′-GGGCGGCCGCCACCATGAATGACCAGTATCACCG-3′ as the forward primer and 5′-GGAAGCTTATCATAGCTCCGTGTCCTCCA-3′ as the reverse primer) from the pCMV-Tag 3B m*Sans* plasmid (Snapgene). It was then inserted into the pAAV.CMV.PI.EGFP.WPRE.bGH vector (Addgene) and packaged into the Anc80L65 capsid. The Sans construct fused to GFP (CMV-GFP-*Sans*-WPRE) was generated using gene synthesis and plasmid subcloning (pAAV.CMV.PI.EGFP.WPRE.bGH) methods (Genscript). Two recombinant viruses, AAV2/Anc80L65-CMV-*Sans*-WPRE and AAV2/Anc80L65-CMV-*GFP*, were produced at the Gene Transfer Vector Core facility (Grousbeck Gene Therapy Center, Schepens Eye Research Institute of Mass. Eye and Ear, Boston, Massachusetts, USA) at a titer of 5.15 × 10^12^ genome copies per milliliter (gc/mL) and 5.5 × 10^12^ gc/mL, respectively. The AAV2/Anc80L65-CMV-GFP-*Sans*-WPRE recombinant virus was produced by ETH Vector Core facility (Zurich, Switzerland) at a titer of 6.2 × 10^12^ gc/mL.

### Animals.

All studies were performed in mice (both male and female) with a mixed C57BL/6J genetic background. The generation and characterization of *Ush1g-*knockout mice has been described elsewhere (13). Animals were housed in the Institut Pasteur animal facilities, accredited by the French Ministry of Agriculture for experiments on live mice.

*Ush1g* recombinant animals were genotyped by means of 2 PCR amplifications, with the oligo-fw1 (5′-GGCCTCGAAGAAGATCCTG-3′) and oligo-rev (5′-GGCAAGTCAAAGGATCAGAT-3′) primers used to detect the wild-type allele (460 bp amplicon) and the oligo-fw2 (5′-CAGTTTCCCCATGTTGATCACCAAC-3′) and oligo-rev primers used to detect the presence of an allele with a deletion of the *Ush*1g exon 2 (332 bp amplicon) and the wild-type (1,964 bp amplicon) allele.

### Vector delivery to the inner ear.

All surgical procedures and viral injections were performed in a biosafety level 2 laboratory. Mice were anesthetized with isoflurane (4% for induction and 2% for maintenance). At the start of surgery, a subcutaneous injection of an analgesic, meloxicam (Metacam, 0.2 mg/kg/d), was administered for pain relief, together with a subcutaneous injection of local anesthetic (lidocaine, Laocaïne, 5 mg/kg) in the retro-auricular region. The anesthetized animal was placed on a heating pad throughout the procedure and until the mouse was fully awake. Intracochlear injections were performed as described by Akil et al. (5). The left and/or right ear was approached via a retro-auricular incision. After dissection of the cervical muscles, the otic bulla was exposed and punctured with a 25-gauge needle. The opening was expanded as necessary with forceps to visualize the stapedial artery and the RWM. A glass pipette was used to puncture the center of the RWM and the viral solution (2 μL viral vector) was then injected through the RWM with a pump system coupled to a glass micropipette. The RWM niche was rapidly sealed after removal of the pipette, with a small plug of muscle secured with a small drop of biological glue (Vetbond 3M) placed on the muscle to prevent leakage from the round window and with a small plug of fat to close the opening of the bulla.

### Immunofluorescence.

The inner ear was perfused with 4% paraformaldehyde in PBS for 45 minutes at 4°C, and the cochlea and vestibular organs were then microdissected and rinsed 3 times in PBS for 10 minutes each. The samples were incubated for 1 hour at room temperature in PBS supplemented with 20% normal horse serum and 0.3% Triton X-100 and were then incubated overnight in PBS with primary antibodies: chicken anti-GFP (1:500; catalog 13970, Abcam) and rabbit anti-myosin VI (1:200) (34). The next day, the samples were rinsed 3 times in PBS for 10 minutes each and incubated for 1 hour at room temperature with the following secondary antibodies: ATTO-488–conjugated goat anti-chicken IgG antibody (1:500 dilution; catalog A11039, Thermo Fisher Scientific) and ATTO-550–conjugated goat anti-rabbit IgG antibody (1:500 dilution; catalog 43328, Sigma-Aldrich). The organ of Corti and vestibular organs were mounted in Fluorsave (Calbiochem). Images were captured with a Zeiss LSM-700 confocal microscope equipped with a Plan Apo-chromat 63×/1.4 N.A. oil immersion lens (Carl Zeiss).

### Hair cell counting.

Transduction rates in the treated ears were calculated by dividing the number of hair cells immunolabeled for GFP by the total number of IHCs, OHCs, and VHCs immunolabeled for myosin VI. Counts were obtained for the apical, middle, and basal regions of the cochlea.

### Scanning electron microscopy.

The inner ear was fixed by incubation in 2.5% glutaraldehyde in 0.1 M phosphate buffer (pH 7.3) for 2 hours at room temperature. The cochlea and vestibular organs were microdissected. The samples were then incubated in 5 alternating baths (OTOTO) of 1% osmium tetraoxide (O) and 0.1 M thiocarbohydrazide (T). The samples were rinsed and dehydrated in a graduated series of ethanol solutions (35%, 50%, 70%, 85%, 95%, and 100%) and then dried to critical point with hexamethyldisilazane. The samples were analyzed in a Jeol JSM6700F-type field emission scanning electron microscope operating at 5 kV. Images were captured with a charge-coupled camera (SIS Megaview3, Surface Imaging Systems).

### Hair-bundle analysis.

Scanning electron microscopy images were analyzed with Photoshop CS6. The length of the stereocilia was determined for the tallest row of IHCs, OHCs, and VHCs. The stereocilia in the tallest row in IHCs and OHCs and the middle row in VHCs were counted. Finally, the diameter of the largest stereocilium in the hair bundle was measured for VHCs.

### Audiological tests.

ABRs to sound stimuli were recorded and analyzed as previously described (4). ABRs to sound stimuli were recorded at least 15 days after injection. Briefly, the mice were anesthetized by intraperitoneal injection of a mixture of ketamine hydrochloride (100 mg/kg) and 2% xylazine hydrochloride (10 mg/kg) and placed in a sound-proofed chamber. Subdermal needle electrodes were placed at the vertex, the ipsilateral mastoid (reference), and on the back (ground). Pure-tone stimuli (bursts) were used at frequencies of 5, 10, 15, 20, 32, and 40 kHz. The hearing threshold was defined as the lowest-level stimulus for which ABR peaks for waves I–V were clearly defined and repeatedly present upon visual inspection.

### Behavioral analysis.

Various behavioral tests were performed to assess vestibular deficits (24). We carefully observed mice to look for circling behavior or head tossing. The trunk curl test was then performed, with the mouse held by its tail; the test was considered successful if the mouse reached a horizontal landing surface (score of 1), partially successful if the mouse almost reached the horizontal surface (score of 0.5), and unsuccessful if the mouse curled its trunk toward its tail (score of 0). The contact righting test was performed by placing the mouse in a transparent tube and determining whether it could right itself rapidly when the tube was rotated 180° (score of 1), whether the rollover occurred but more slowly (score of 0.5), or whether the mouse failed to right itself (score of 0). In the platform test, the time spent by the mouse on a platform (7 cm × 7 cm wide and at a height of 29 cm) before falling off was determined over a period of 1 minute. The test was repeated 3 times and the mean time spent on the platform over the monitoring period was determined. We scored the swimming ability of each mouse in a container (at least 15 cm deep) filled with water at 22°C–23°C over a period of 1 minute as follows: 0, if the mouse swam correctly, with the body elongated and the tail propelling in a flagella-like motion; 1, if swimming was irregular (vertical swimming, swimming in a circle, swimming on the side, swimming in an unbalanced manner); 2, if the mouse remained in an immobile floating position; and 3, if the mouse was drowning.

Finally, mouse behavior was recorded and analyzed with EthoVision XT video tracking software (Noldus Information Technology). The mouse was placed in its cage and tracked by video over a period of 3 minutes. The location and movement of the tip of the noise, the central point of the animal, and the tail base were determined. The distance covered (in cm) and the number of rotations during 3 minutes were recorded.

### VOR analysis.

The experimental set-up, apparatus and data acquisition mechanisms were as previously described (35, 36). For pupil position recording with the head fixed, a head post was implanted in the skull of the animal at least 48 hours before vestibular exploration, following the methodology previously described (37). All eye movements were recorded in the dark with an infrared video system (ETL-200, ISCAN), using noninvasive video-oculography methods to record pupil and corneal reflection position. Mice were head-fixed in an approximately 30° nose-down position to align the horizontal canals with the yaw plane. Myosis was induced by the topical application of 2% pilocarpine 10 minutes before the experiment. The recorded eye and head position signals were sampled at 1 kHz, digitally recorded (CED power1401 MkII) with Spike 2 software, and exported into the Matlab programming environment for off-line analysis (The MathWorks). Videonystagmography was performed to record spontaneous eye movements without vestibular stimulation and eye movements during sinusoidal rotation for the horizontal angular VOR and static head tilt roll for the OCR or utriculo-ocular reflex. Briefly, angular VOR was tested during horizontal sinusoidal rotation of the turntable (at 0.2, 0.5, 0.8, 1, and 1.5Hz; peak velocity, 30°/s), with gain and phase analyzed. The gain was defined as the amplitude of the eye (response) rotation over the amplitude of the head (stimulus) rotation. As the head of the animal was fixed to the rotating table, the movements of the head and the table were identical. The phase was the temporal shift between the eye and table rotations, expressed in degrees as a ratio of the sinusoidal cycle (2 pi) (38). The static OCR tests specifically tested the static utriculo-ocular reflex. Vertical pupil position was measured as a function of head tilt angle with the platform tilted to different roll positions, at 10, 20, 30, and 40 degrees to the right and left alternately. Measurements were made in a static position over at least 15 seconds to identify the stable pupil position. The vertical eye angle was then calculated from the raw vertical pupil position (39). The OCR gain was calculated as the slope of a linear regression line for both variables (vertical eye angle and head tilt in degrees).

### Statistics.

Values are presented as *n* (%) or mean ± SEM. All statistical analyses were performed with Prism 6 (GraphPad Software) after use of the D’Agostino & Pearson omnibus normality test to determine whether the data were normally distributed. For quantitative data, Mann-Whitney *U* tests or Student’s 2-tailed *t* tests were performed for 2-group comparisons, and 1- or 2-way ANOVA was used for comparisons of more than 2 groups, with systematic correction for repeated measures. Two-tailed Fisher’s exact tests and χ^2^ tests were used for qualitative data. Differences were considered to be significant if *P* values were less than 0.05.

### Study approval.

Animals were used in accordance with the European Communities Council Directive 2010/63/EU. All procedures were approved by ethics committees of the Université Paris Cité, INSERM, and Institut Pasteur.

### Data availability.

All data reported in this paper are available from SS upon request. Data point values for all graphs are found in the Supporting Data Values file.

## Author contributions

GL designed and performed experiments on mice (injections, auditory and vestibular assessments, immunofluorescence, scanning electron microscopy), analyzed data, and wrote the manuscript. CC performed experiments on mice (injections, auditory and vestibular assessments). FS conducted experiments (VOR recordings), analyzed data, and wrote the manuscript. VM conducted experiments (scanning electron microscopy). LA performed experiments on mice (auditory and vestibular assessments). BP performed experiments on mice (auditory and vestibular assessments, immunofluorescence). JBDM analyzed data. MJL reviewed the manuscript. MB designed VOR experiments, secured funding and ethical authorizations, analyzed data, and wrote the manuscript. CP reviewed the manuscript. SS designed the research study, analyzed data, and wrote the manuscript.

## Supplementary Material

Supplemental data

Supporting data values

## Version 1 01/09/2024

In-Press Preview

## Version 2 02/08/2024

Electronic publication
