# Supplementary material for: Extended time frame for restoring inner ear function through gene therapy in Usher1G preclinical model
Source: JCI Insight. 2024 Feb 8;9(3):e169504. doi: 10.1172/jci.insight.169504 (PMC10967394; doi:10.1172/jci.insight.169504)
Supplement: Supplemental data [file jciinsight-9-169504-s177.pdf]

Supplementary material

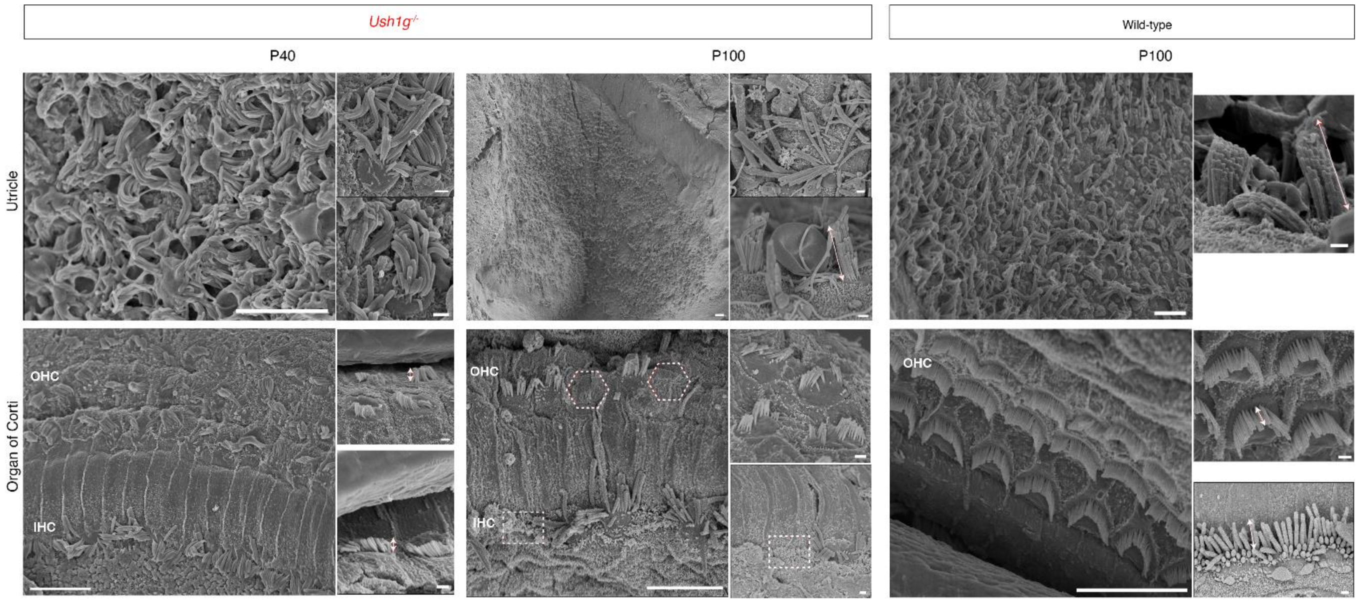

Supplementary Figure 1. **Morphostructural analyze of hair cell bundle in the utricle and organ of Corti in adulte *Ush1g*$^{-/-}$ and wild type mice**. Low and intermediate magnification scanning electron micrographs showing the progressive degeneration of the hair bundle and the hair cells. At P40, in both utricle (uper panels) and in the cochlea (lower panels), the hair bundles are disorganized, and lacking their normal staircase pattern. Also, the length of OHC and IHC stereocilia is significantly reduced (white two-headed arrows). At P100, hair cell loss in both utricle and cochlea is taking place. The low magnification view of the utricle illustrates a sensory epithelium almost devoid of hair cells, whereas the view of the organ of Corti exhibits notable hair loss (oulined in white). Finally, the VHC hair bundle displays either a complete disorganized aspect with large and long stereocilia, or a quite well-organized aspect but with a reduced length. Scale bars: low magnification micrographs 10 µm, intermediate magnification micrographs 1 µm.

	Untreated *Ush1g*$^{-/-}$ mice	Treated *Ush1g*$^{-/-}$ mice	Wild-type mice
OHC stereocilia length (µm)	1.3±0.08 [1.1-1.7] *(n=8)*	2.1±0.07 [1.72-2.4] *(n=9)*	1.8±0.08 [1.6-2.2] *(n=6)*
OHC - Number of stereocilia	8±0.5 [7-11] *(n=8)*	16±0.4 [14-18] *(n=10)*	29±1.4 [22-32] *(n=8)*
IHC stereocilia length (µm)	3.9±0.35 [3.2-4.8] *(n=4)*	3.3±0.27 [2.8-4.1] *(n=4)*	3±0.06 [2.9-3.2] *(n=4)*
IHC - Number of stereocilia	4±0.4 [3-5] *(n=6)*	9±0.4 [7-10] *(n=8)*	10±0.4 [9-11] *(n=8)*
VHC stereocilia length (µm)	7.1±0.73 [2.40-11.06] *(n=11)*	8.6±0.43 [7.20-10.70] *(n=9)*	7±0.39 [5.45-8.40] *(n=8)*
VHC stereocilia width (µm)	0.6±0.08 [0.28-1.24] *(n=13)*	0.3±0.01 [0.25-0.36] *(n=9)*	0.2±0.01 [0.22-0.26] *(n=4)*
VHC – Number of stereocilia	5±0.5 [5-6] *(n=2)*	5±0.2 [5-6] *(n=7)*	6±0.2 [5-7] *(n=8)*

Supplementary Table 1. **Assessment of stereocilia size of OHC, IHC, and utricular VHC using scanning electro-micrographs at P112.** Inner ear hair cell stereocilia of wild-type mice, untreated *Ush1g*$^{-/-}$ mice, and treated *Ush1g*$^{-/-}$ mice with AAV2/Anc80L65-*Sans* were analyzed. Measures are: stereocilia height of the higher row, number of stereocilia of one row (higher row for OHC and IHC, central row for VHC), width of the larger stereocilia in the bundle for VHC.

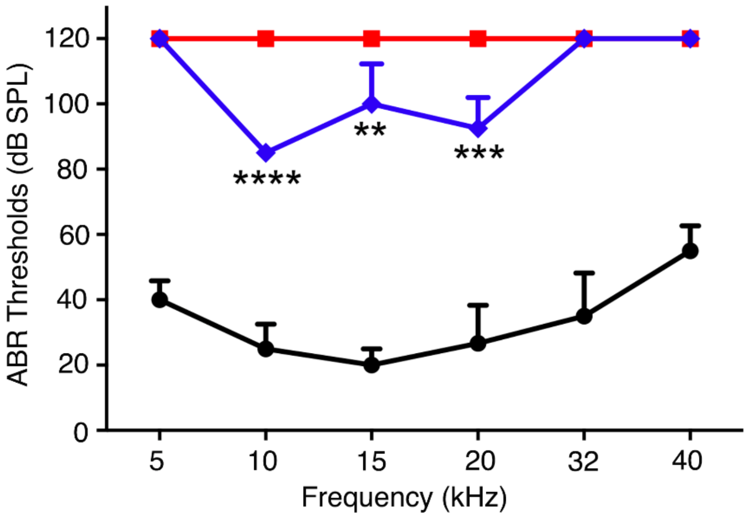

Supplementary Figure 2. The hearing recovery is still present on p60 after a single injection performed through the round window membrane of *Ush1g*$^{-/-}$ mice between P12 and P21. Significant improvement of thresholds is observed at 10, 15, and 20 kHz for treated *Ush1g*$^{-/-}$ mice (n=4) compared to untreated *Ush1g*$^{-/-}$ mice (n=6) (p=0.0001, ANOVA).

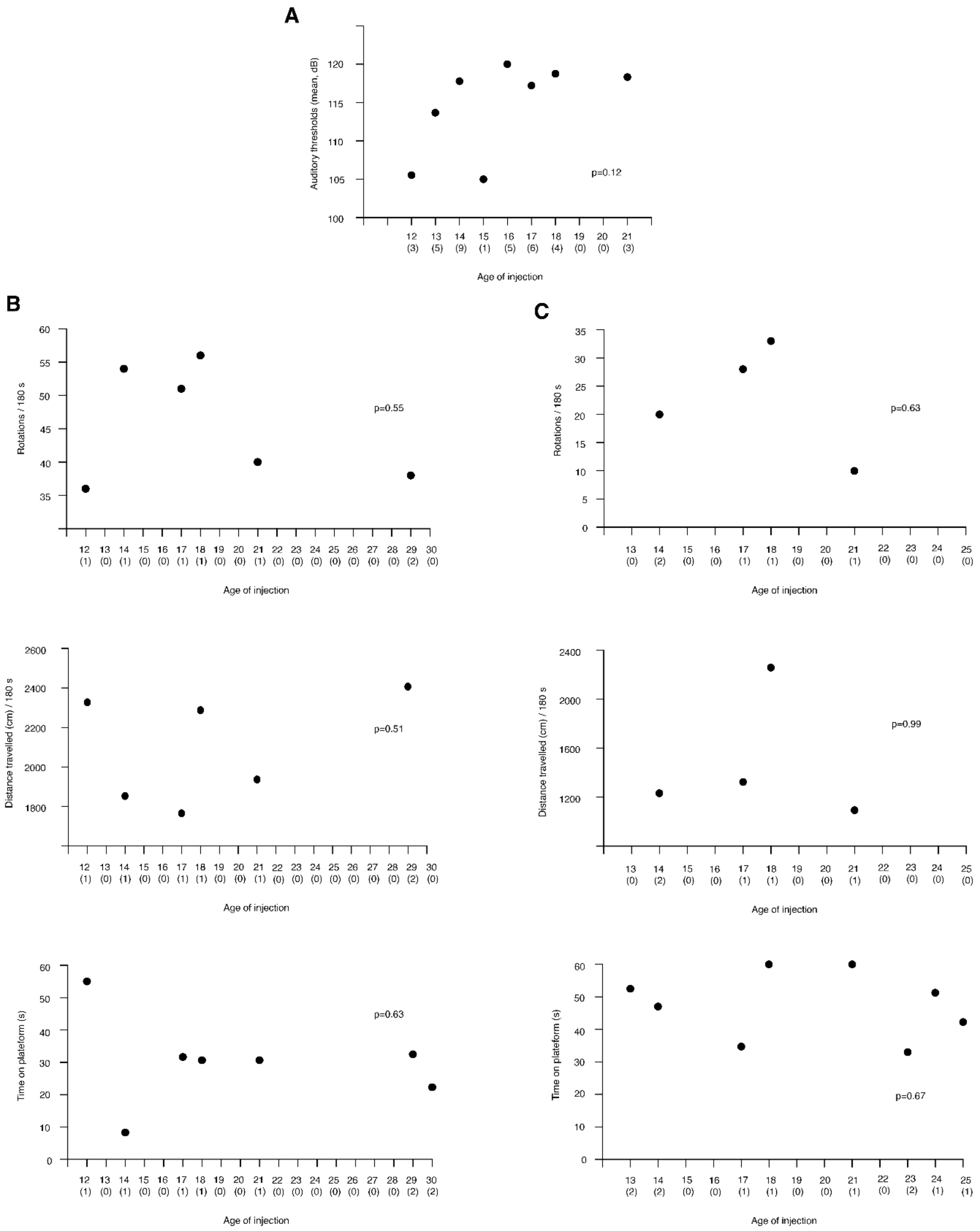

Supplementary Figure 3. Auditory and vestibular function following intracochlear gene therapy depending on the age of injection in *Ush1g*$^{-/-}$ mice. The x-axis denotes both the age and the number of the treated mice. **(A)** Mean of auditory thresholds obtained at 5, 10, 15, 20, 32, and 42 kHz measured at P40. **(B, C)** Locomotor tests (time on platform and distance traveled) performed at

P40 in *Ush1g*$^{-/-}$ mice treated unilaterally **(B)** and bilaterally **(C)**. No correlation was found between auditory or vestibular recovery and exact age of injection (logistic regression test).

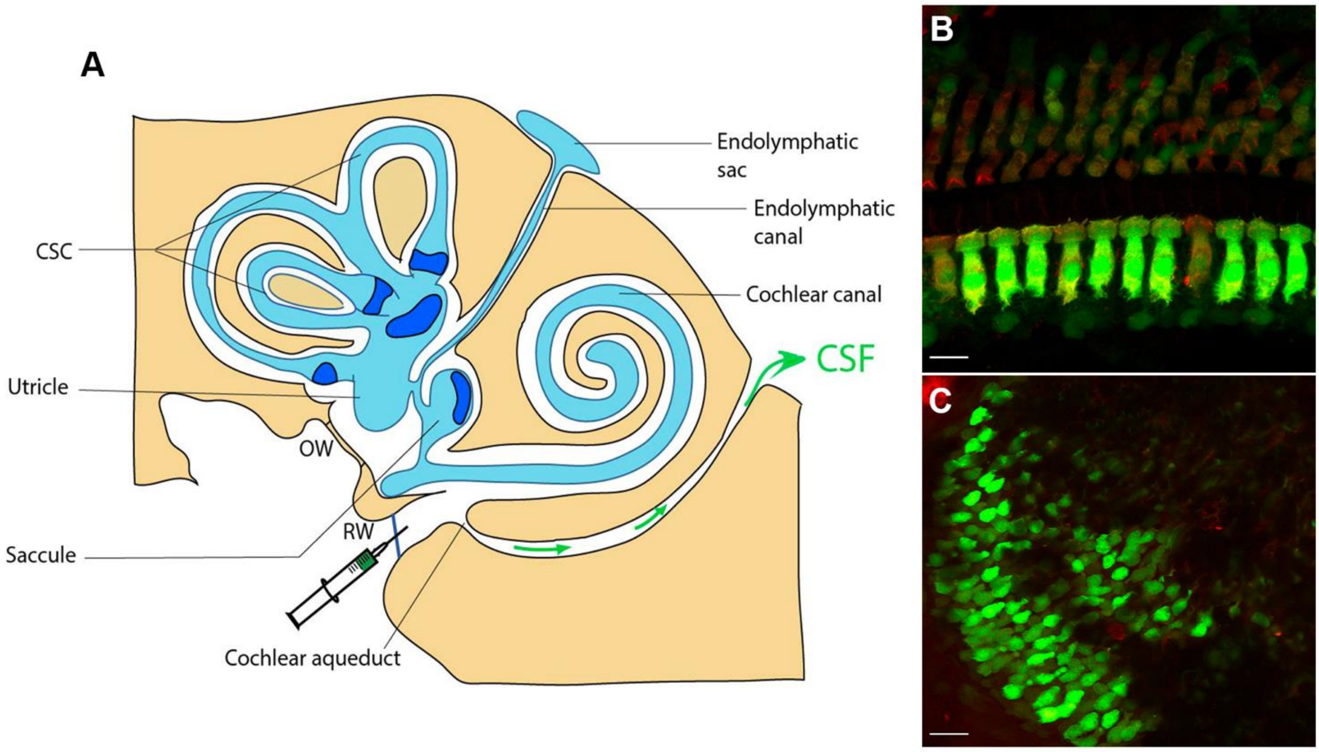

Supplementary Figure 4. Unilateral intracochlear injection of AAV2/Anc80L65-GFP through the round window membrane (RWM) during the neonatal stage (P2) resulted in the transduction of sensory hair cells in both ears (A) The green arrows schematically represent the vector diffusing through the cochlear aqueduct, connecting the perilymphatic space and the cerebrospinal fluid (CSF), and subsequently reaching the contralateral ear through its respective cochlear aqueduct. **(B)** The Organ of Corti and **(C)** utriculi macula in the contralateral ear of a neonatal-stage wild-type mouse, demonstrating transduction of the vast majority of cochlear hair cells. (GFP in green, Myosine VI in red). Scale bars: upper 10 µm, lower 20 µm.

	Untreated *Ush1g*$^{-/-}$ mice (n=10)	Unilaterally treated *Ush1g*$^{-/-}$ mice (n=9)	Bilaterally treated *Ush1g*$^{-/-}$ mice (n=11)	Wild-type mice (n=10)
Time spent on platform (s, mean±SEM [range])	12±2.4 [0-22]	30±5.9 [1-55]	43±4.8 [13-60]	56±2.4 [41-60]
Score on the swimming test				
0	0	0	0	10
1	0	2	3	0
2	0	0	3	0
3	10	7	5	0
Score on the trunk curl test				
0	10	1	2	0
0.5	0	5	3	0
1	0	3	6	10
Score on the contact righting test				
0	10	2	2	0
0.5	0	2	4	0
1	0	5	5	10
Circling behavior				
No circling	0	4	6	10
Circling	10	5	5	0
Head tossing				
No tossing	0	5	7	10
Tossing	10	4	4	0

Supplementary Table 2. Outcomes of behavioral tests performed at P40 in untreated *Ush1g*$^{-/-}$, as well as in unilaterally and bilaterally treated *Ush1g*$^{-/-}$, and wild-type mice.

Score of the swimming test : score of 0 if the mouse swam correctly, with the body elongated and the tail propelling in a flagella-like motion; 1 if swimming was irregular (vertical swimming, swimming in a circle, swimming on the side, swimming in an unbalanced manner); 2 if the mouse remained in an immobile floating position; 3 if the mouse was drowning.

Score of the trunk curl test : the test was considered successful if the mouse reached a horizontal landing surface (score of 1), partially successful if the mouse almost reached the horizontal surface (score of 0.5), and unsuccessful if the mouse curled its trunk toward its tail (score of 0).

Score of the contact righting test : score of 1 if the mouse could right itself rapidly in a tube rotated at 180°, score of 0.5 if the rollover occurred but more slowly, score of 0 if the mouse failed to right itself.

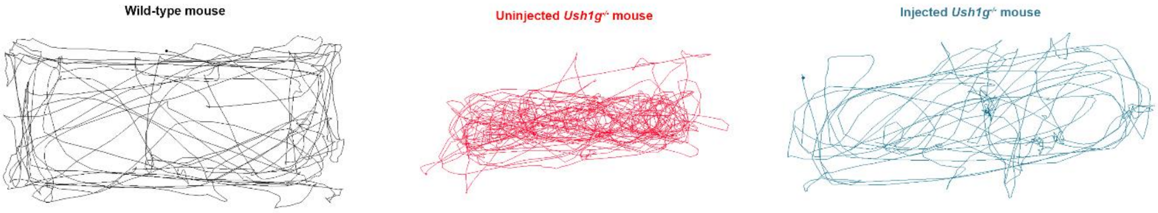

Supplementary Figure 5. Video-tracking in an open-field chamber over a period of 3 minutes of p40 wild-type, *Ush1g*$^{-/-}$, and bilaterally injected *Ush1g*$^{-/-}$ mice on p14. The behavior of bilaterally treated mice closely resembled that of control mice as they explored the field.

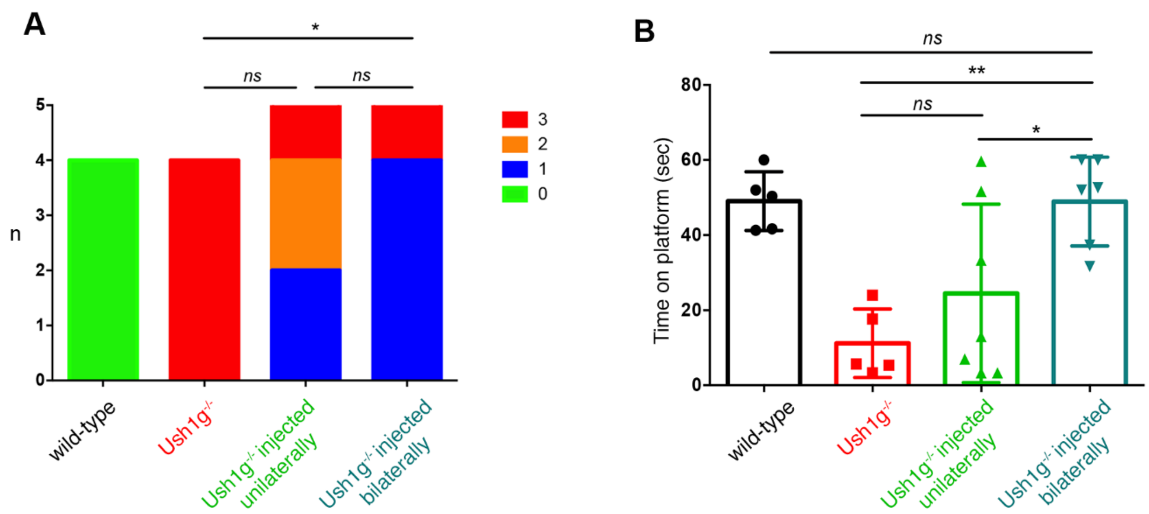

Supplementary Figure 6. Vestibular function recovery in *Ush1g*$^{-/-}$ mice after intracochlear AAV2/Anc80L65-*Sans* delivery at a mature stage is still observed at P100. (A) A significant difference was observed in the swim test scores of bilaterally injected *Ush1g*$^{-/-}$ mice when compared to non-injected *Ush1g*$^{-/-}$ mice (p=0.03, Mann-Whitney test), 4/5 of the injected mice displayed an irregular swim whereas all non-injected mice started drowning. **(B)** The bilaterally injected *Ush1g*$^{-/-}$ mice spent significantly more time on the platform compared to untreated *Ush1g*$^{-/-}$ mice (p=0.004, Mann-Whitney test) and unilaterally injected *Ush1g*$^{-/-}$ mice (p=0.04, Mann-Whitney test), with no significant difference with the wild-type mice.

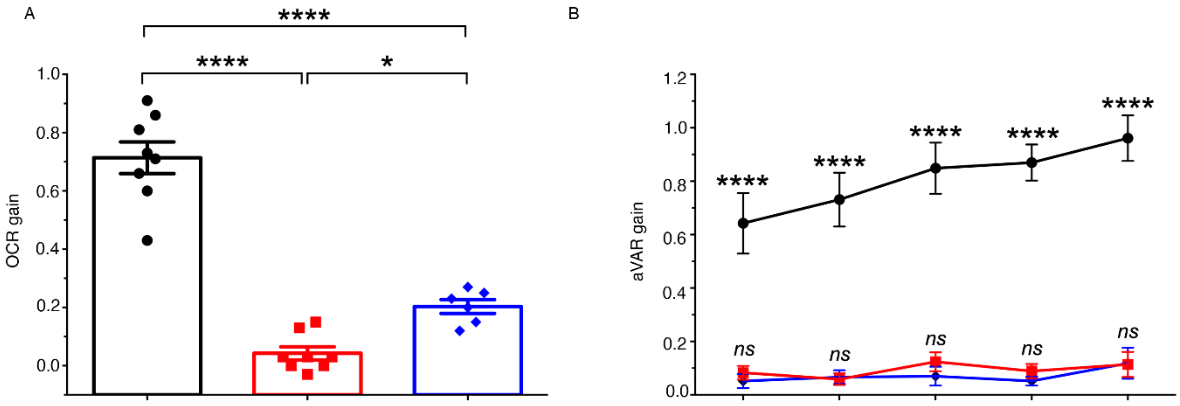

Supplementary Figure 7. Measures of the angular vestibulo-ocular response gain (VOR) and the ocular counter roll response gain (OCR) using nystagmography at P140. (A) Significant improvement of the OCR gain after bilateral administration of AAV2/Anc80L65-*Sans* at mature stage in *Ush1g*$^{-/-}$ mice compared to untreated mice (respectively 0.2±0.02, n=6 – *blue*, and 0.04±0.02, n=8 - *red*, p=0.03, One-way ANOVA), remaining under the gain measured for the wild-type mice (*black*, 0.7±0.05, n=8 p<0.0001, One-way ANOVA). **(B)** No improvement of the angular VOR gain after bilateral injection (*blue*) compared to the *Ush1g*$^{-/-}$ untreated mice (*red*).
